# Kyphectomy and sliding growing rod technique in patients with congenital lumbar kyphosis deformity with myelomeningocele

**DOI:** 10.1186/s13018-024-04577-3

**Published:** 2024-02-03

**Authors:** Muhammed Enes Karataş, Yusuf Bayram, Halid Şafak, İlyas Kar, Necdet Sağlam, Bekir Yavuz Uçar

**Affiliations:** 1 Department of Orthopaedics and Traumatology, Kartal Dr.Lütfi Kırdar City Hospital, Istanbul, Turkey; 2 Department of Orthopaedics and Traumatology, Hisar İntercontinental Hospital, Istanbul, Turkey; 3 Department of Orthopaedics and Traumatology, Gumushane State Hospital, Istanbul, Turkey; 4 Department of Orthopaedics and Traumatology, Umraniye Training and Research Hospital, Istanbul, Turkey; 5 Department of Orthopaedics and Traumatology, Istanbul Medipol University, Istanbul, Turkey

**Keywords:** Spina bifida, Myelomeningocele, Kyphosis, Kyphectomy, Spine, Sliding growing rod

## Abstract

**Objective:**

Neural tube defects are the most common congenital disorders after cardiac anomalies. Lumbar kyphosis deformity is observed in 8–15% of these patients. This deformity severely limits the daily lives of these patients. In our study, we aimed to correct the kyphosis angle of the patients with lumbar kyphosis associated with myelomeningocele (MMC) and allow them to continue their growth without limiting their lung capacity by applying kyphectomy and sliding growing rod technique.

**Patients and methods:**

In this study, we retrospectively evaluated 24 patients with congenital lumbar kyphosis deformity associated with MMC, aged between 4 and 9 years, and who applied to Umraniye Training and Research Hospital between the dates of 2018 and 2021. We evaluated preoperative and postoperative kyphosis angles, correction rates, bleeding during operations, operation time, level of instrumentation, number of the resected vertebrae, initial levels of the posterior defects, duration of hospital stays, annual lengthening, and weight of the patients.

**Results:**

Mean age was 5.04 (between 4 and 9). Mean preoperative and early postoperative kyphosis angles were 129.8° (87–175°) and 0.79° (− 20–24°), respectively. The kyphotic deformity correction rate was 99.1%. A difference was found regarding kyphosis measurements between preoperative and early period values (*p* < 0.05). The annual height lengthening of patients was calculated as 0.74 cm/year and 0.77 cm/year between T1–T12 and T1–S1, respectively. Mean preoperative level of hemoglobin (Hgb) was 11.95, postoperative Hgb value was 10.02, and the decrease was significant (*p* < 0.05). In terms of complications, 50% (12) had broken/loosen screws, 50% (12) had undergone debridement surgery, 37.5% (9) had vacuum-assisted closure therapy, and 33.3% (8) had to get all of their implants removed.

**Conclusion:**

We believe that our sliding growing rod technique is a new and updated surgical method that can be applied in these patient groups, facilitating the life, rehabilitation process, and daily care of MMC patients with lumbar kyphosis. This technique seems to be a safe and reliable method which preserves lung capacity and allows lengthening.

## Introduction

Spina bifida (SB) occurs when arcus vertebrae lack a spinous process and stay incomplete as a result of the failure of neural tube closure which is expected to occur in the 4th week of the fetal development. SB is classified into two groups as open (spina bifida aperta/cystica) and closed (spina bifida occulta). The most common and severe form of SB is myelomeningocele (MMC), the most severe form of all birth defects compatible with life [1].

The most common vertebral deformities in MMC patients are scoliosis, kyphosis, and sacral agenesis [2]. The incidence of kyphosis in these patients is between 8 and 20%. The angle is over 80°, and there is an annual increase between 6 and 12°. Depending on the type of their deformity, patients might develop following disorders; pulmonary disorders, poor postural control, congestion in abdominal organs, and ulcerative pressure sores either over kyphotic region due to vertebral deformity or around rib due to scoliotic posture which can lead to osteomyelitis.

Patients with rigid lumbar kyphosis have a clinical characteristic image. These patients develop extension deformities as they force cervical extension to keep their balance and horizontal view. They are not able to lie flat as a result of the development of kyphosis. They also lose their movement abilities in upper extremities in time if they are left untreated.

Our treatment target for patients with MMC is a balanced spine. Thoracic deficiency can also be prevented in these patients with a maintained sagittal balance in the early term. Decubitus sore on the sacral area can be avoided by the fixation of pelvic obliquity. One of the main aims of the treatment is to prevent the abdominal congestion which occurs due to lumbar kyphosis. The principles of deformity surgery in growing spine can also be applied to patients with SB with MMC defects. This patient group is reported to get benefited from the growing rod systems which are also used in corrective pediatric deformity surgeries [3].

In this study, we aim to correct congenital lumbar kyphosis which is common in MMC patients with SB through kyphectomy and posterior instrumentation and support the growing and developing spine without spinal fusion surgery. So, we aim to maintain a balanced spine while also protecting pulmonary capacity in patients. With this aim in mind, we also evaluated the possible complications along with clinical and radiological outcomes of our operations.

## Method

We retrospectively evaluated the defects of 24 MMC patients with congenital lumbar kyphosis deformities between the years 2018 and 2021 in the Umraniye Training and Research Hospital Orthopedic and Traumatology Clinic.

We included MMC patients between 4 and 9 years of age with accompanying congenital lumbar kyphosis only with defects at the T6 level and below and who were operated with sliding growing rod technique and kyphectomy. We excluded patients with insufficient medical history, who stopped having treatment in our clinic and continued their treatment in another one, who underwent one-sided sliding growing rod technique and fusion surgery, and revision patients whose previous operations were unsuccessful. We also included patients with a follow-up period of at least 3 years and excluded patients with a follow-up period of under 3 years. The study complies with the principles of Declaration of Helsinki as a statement of ethical principles for medical research involving human subjects and approved by Umraniye Training and Research Hospital Ethics Committee with an approval number of 00149248223.

AP and lateral graphs of patients’ full-length spine were preoperatively taken in sitting position and recorded in a single cassette, and similar graphs were also taken during follow-ups. The patients were operated by three spine surgeons, two of which cooperated in all operations.

Patients’ lengthening between T1–T12 and T1–S1 vertebrae was compared and evaluated through AP and lateral X-rays and their early postoperative graphs and last graphs.

All patients were first mobilized in bed while still in the ward under the supervision of our physiotherapist before discharge. After the wound healed, our patients were mobilized on a walking belt with knee–ankle–foot orthosis (KAFO).

All patients had motor and sensory deficits below the vertebral level at which MMC had first started. Existing kyphotic deformity negatively affected the patients’ sitting balance and prevented them from lying flat on their backs and caused some of them to have pressure ulcers and exposed them to soft tissue infections.

### Surgical technique

**Fig. 1:**
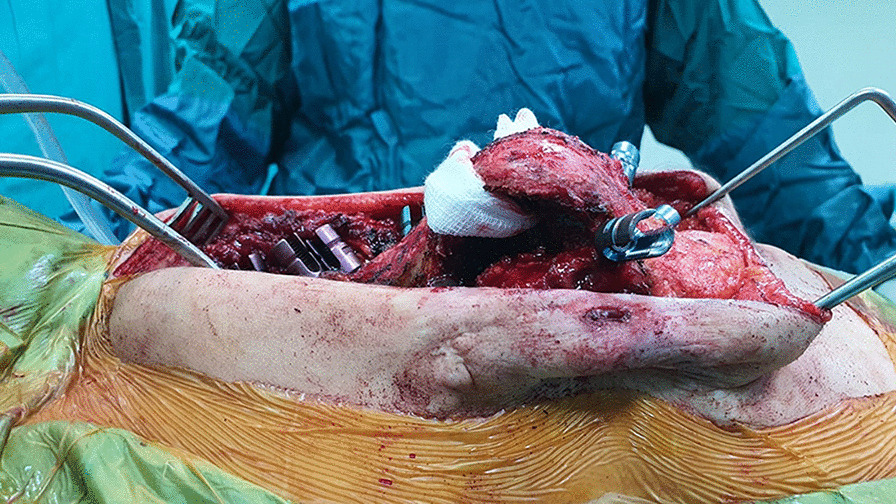
Getting lumbar kyphosis area ready for kyphectomy

**Fig. 2:**
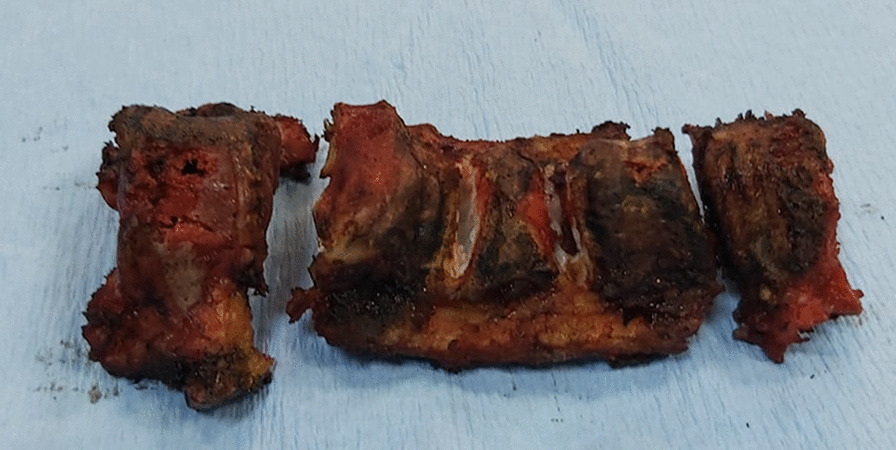
Corpus vertebrae after kyphectomy

**Fig. 3:**
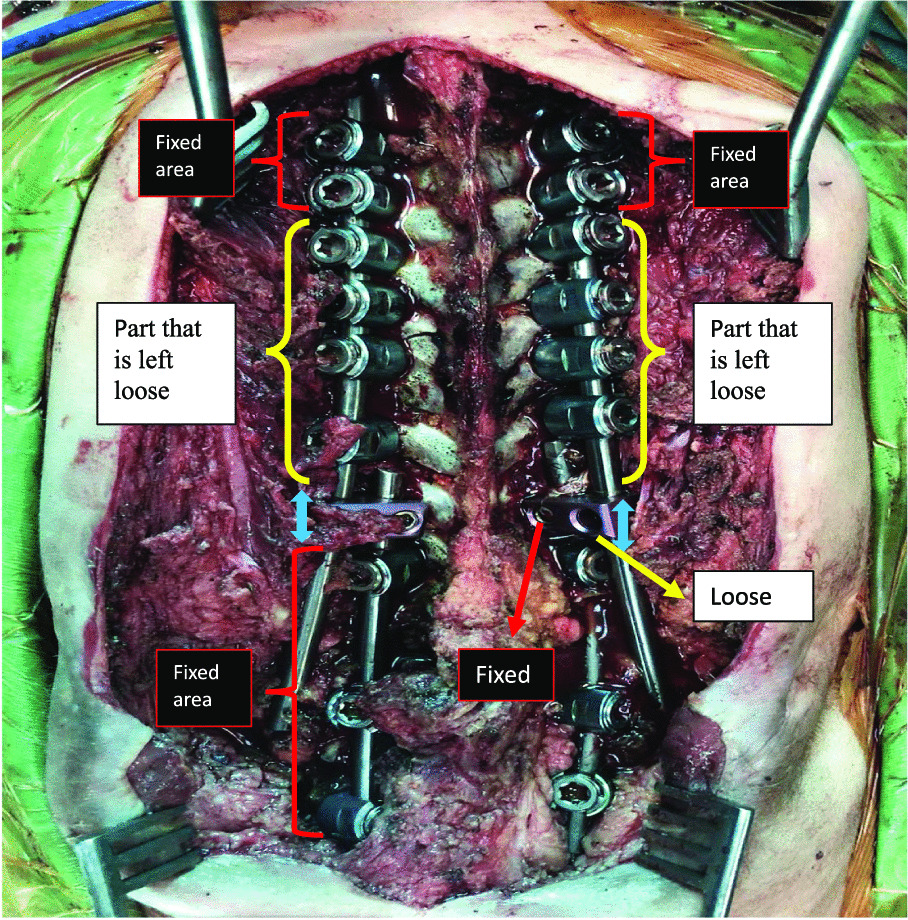
Sliding growing rod technique. Domino connectors were placed in lumbar area. The most proximal and distal screws were fixated and fused. The rest of the system was not locked, we expect growth between T4 and T9

Patients were placed in prone position. First-generation cephalosporin (30/40 mg/kg) prophylaxis was administered 30 min. Before surgery, an additional dose of antibiotic was applied in surgeries over 4 h. Paraspinal muscles and osseous structures were subperiosteally dissected through posterior incision, and deformity was exposed. All patients underwent kyphectomy and posterior instrumentation for the application of sliding growing rod technique (Fig. 1). The surgical technique for the dissection of the anterior parts of the vertebrae was carried out at utmost attention. Anterior longitudinal ligaments (ALL) were kept to stay away from anterior vascular structures and used as a barrier in between. Coronal and sagittal balance was achieved along with a strong and durable fixation, and proximal levels of T2–T3 vertebrae were reached to eliminate the possibility of failure. Yet again, the fixation was strengthened at the bottom level with neat and regular iliac screws. Upon completing posterior instrumentation, we applied kyphectomy while protecting dura. Vertebrae were identified for corpectomy so as to provide bone-to-bone fusion (Fig. 2). Intervertebral disks were totally cleaned in upper and lower endplates of the vertebrae in order for them to provide bone fusion, and this area was applied compression. Rods were implanted after corpectomy. A total of four rods (two of then were placed above and two of them were placed below the fusion level) were implanted with dominos. Pedicle screws in the fusion area were locked to the rod together with the pedicle screws that belong to two proximal vertebrae. The nuts of the other pedicle screws were left loose to provide a sliding rod. We applied our growing protection technique by allowing the system to grow in the region starting from kyphectomy area to two most proximal vertebrae [4]. All patients were applied iliopelvic fixation both to prevent lumbosacral instability and maintain balance while sitting (Fig. 3).

### Statistical method

Social Sciences (SPSS) Mac Version 25 (IBM, Armonk, NY, USA) software was used in the evaluation of the research data. Kolmogorov–Smirnov was used to determine whether continuous variables were close to normal distribution, and a range of ± 1.5 was considered acceptable for normal distribution. Paired t-test was used for numerical variables when comparing paired dependent groups. Binary group comparisons were conducted with Chi-square test when the conditions were met for categorical variables. A statistical significance level of 95% confidence interval and a *p* value less than 0.05 were accepted as significant.

### Findings

We included a total of 24 patients diagnosed with MMC with congenital lumbar kyphosis pathology between the years 2018 and 2021. Of 24 patients, 58.3% (14) were male, and 41.7% (10) were female. Mean age was 5.04 (between 4 and 9). Mean follow-up period was 53.6 months (between 42 and 70 months). Mean lengthening between T1 and T12 was 0.74 cm/year (between 5.5 and 9), and mean lengthening between T1 and S1 was 0.77 cm/year (between 6 and 9.5).

**Fig. 4:**
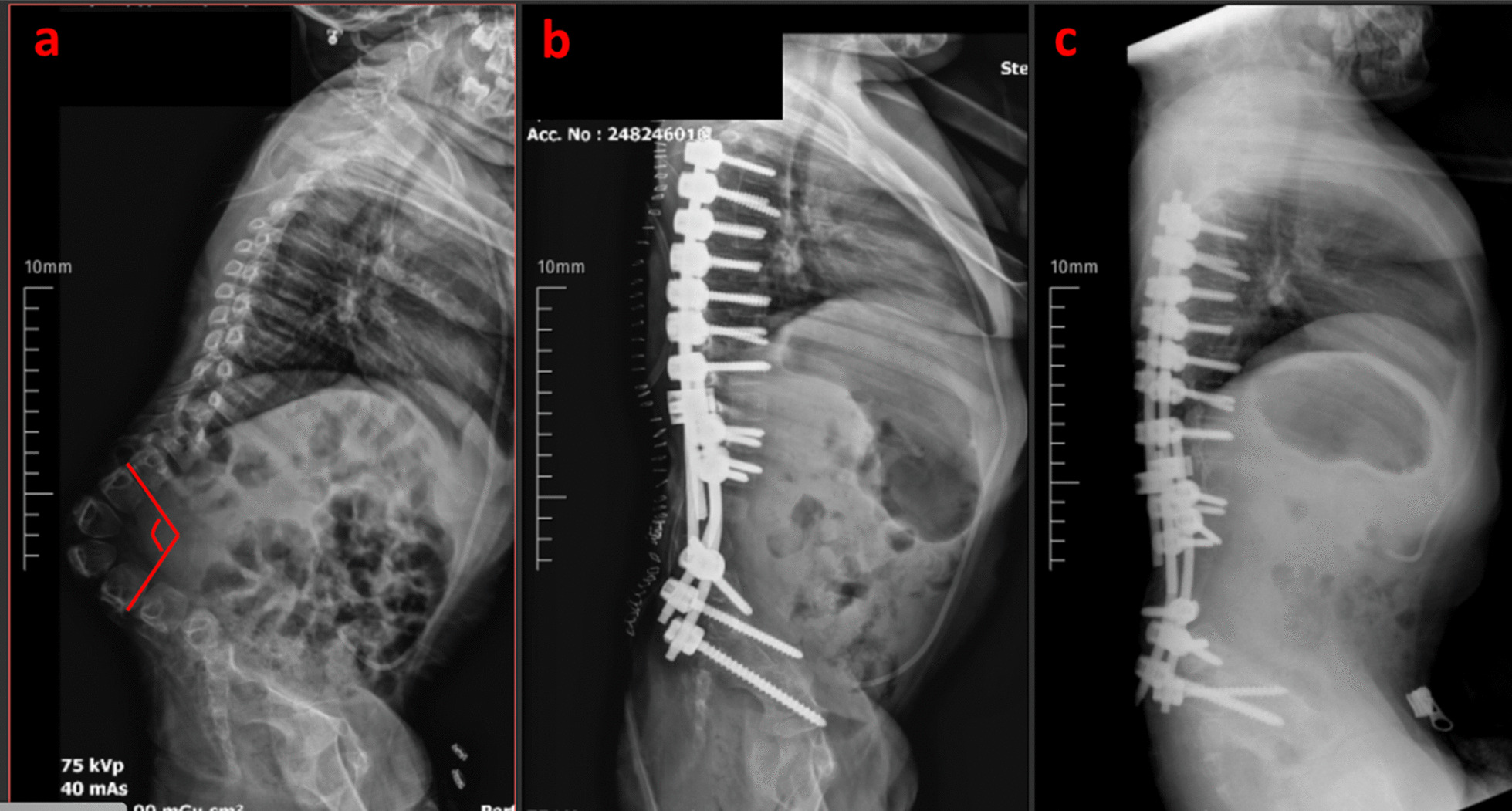
Preoperative and postoperative altered kyphosis angle. **A**. Preoperative kyphosis angle 118°. **B**. Early postop kyphosis angle – 1 °. **C**. Radiograph at last follow-up

Mean preoperative and postoperative kyphosis angles were observed as 129.8° (between 87° and 175°) and 0.79° ± 10.5, respectively. A statistically significant difference was reported between preoperative and postoperative kyphosis angles (*p* < 0.05). Kyphotic deformity correction rate was 99.1% (Table 1) (Fig. 4).

**Table 1:** Preoperative and postoperative kyphosis values of patients

	Mean ± SD	Min–max
Preop Kyph	129.88 ± 26.04	87–175
Postop Kyph	0.79 ± 10.57	(− 20)- 24
Preop–postop Kyph Difference	129.08 ± 29.08	84–177

The mean pelvic tilt of the patients decreased from 48.25° ± 15.3 preoperatively to 22.7° ± 8.8 postoperatively. When these two values were compared, it was determined to be statistically significant (*p* < 0.05) (Table 2). Mean operation time (starting from the first incision to the last suture) was 301.2 (240–390) min.

**Table 2:** Preoperative and postoperative pelvic tilt angles of patients

	Preop	Postop		
	Mean ± SD	Mean ± SD	*t*	*p*
Pelvik tilt angle	48.25 ± 15.3	22.7 ± 8.8	10.7	.000

Patients were applied one unit of erythrose transfusion. Mean preoperative and postoperative hemogram of patients was 11.9 g/dl (9.8–16 g/dl) and 10.02 g/dl (7.7–13.9 g/dl), respectively. Postoperative hemoglobin value was reported to decrease significantly compared to preoperative value (Table 3).

**Table 3:** Comparison of preoperative and postoperative values of Hgb

	Preop	Postop		
	Mean ± SD	Mean ± SD	*t*	*p*
Hemoglobin	11.95 ± 1.46	10.02 ± 1.48	9.855	000

Mean hospitalization rate and mean length of intensive care unit stay of the patients were 28.7 (between 5 and 90) days and 7.08 (2–35) days, respectively.

Regarding comorbidities, all patients (24) had urological conditions, 95.8% (23) had neurological conditions, 37.5% (9) had ophthalmic conditions, 8.3% (2) endocrine conditions, and 8.3% had cardiac disorders.

### Complications

**Fig. 5:**
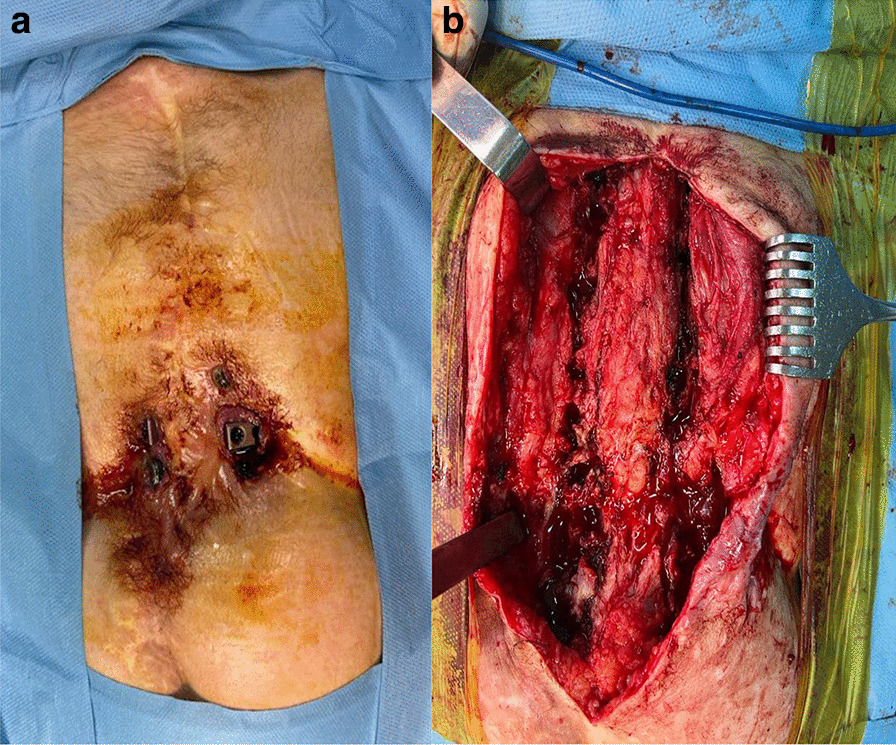
The exposition of the implant. **a** Exposed image of the implants after skin irritation. **b** We removed all implants of a patient who we believed had fusion in their kyphectomy area

Of all the patients, 50% (12) had broken/loosen screws, 50% (12) had undergone debridement surgery, 37.5% (9) had vacuum-assisted closure (VAC) therapy, and 33.3% (8) had the need to get all of their implants removed (Fig. 5). Kyphosis angles were progressed in three out of eight patients who got all their implants removed; however, five of them showed no progression even after 1 year. After the removal of the implants, CT scans revealed that out of eight patients, five had fusion, and three developed pseudoarthrosis.

Of all patients, 58.3% (14) had wound problems or surgical site infection in the postoperative period. No significant difference was reported between the preoperative and postoperative values of C-reactive protein (CRP) (*p* > 0.05).

No statistical difference was observed among incidence periods, preoperative and postoperative hemoglobin rates, length of hospitalization (days), and intensive care unit stay (days) of the patients who underwent debridement and VAC therapies (*p* > 0.05).

## Discussion

Kyphosis in patients with MMC associated with spinal deformity severely affects daily life functions. In these patients, kyphosis often has a progressive course. Sharp kyphosis leads to balance impairments in sitting and soft tissue ulcerations which lead to osteomyelitis. This deformity affects bimanual functions so, when patients use their hands to keep the body upright, they cannot continue their in-hand manipulation (IHM) skills at the same time.

Conservative treatment methods (corsets, modifications of wheelchairs, etc.) are not effective in these patients, they rather increase soft tissue problems [5]. Surgery is nearly the only option and is helpful not only in supporting sitting balance but also in relieving chronical and consistent pressure ulcers. To correct severe kyphosis, some techniques are defined in the literature such as Harrington rods, plaque fixations, Galveston technique, Dunn–McCarty fixation, and Warner and Fackler technique. Lately, defined techniques mostly concentrate on growth supporting systems and the correction of sagittal plane deformity.

Early interventions in MMC patients are essential in preventing the progression of the rigidity of the deformity and reduce the other possible deformities (respiratory problems in particular). In this patient group, kyphosis-associated secondary scoliosis might cause respiratory problems as development of the lungs continues until 8 years of age [6]. There are conflicts in the best possible time for surgery in the literature. Sharard et al. supported the idea of correcting the deformity right after birth while Hall and Poitras did not recommend neonatal kyphectomy due to high rates of failure. They rather claimed that the surgery should be postponed until 3 years of age when morphology of the spine develops and allows a better fixation. According to them, although an early kyphectomy looks tempting due to less severe kyphotic deformity, it might also prevent body from growing and leads to a shorter body in patients who have not completed their skeletal growth yet [7, 8]. Due to the same reasons and possible complications, we also agree that kyphectomy is not convenient for patients under 3 years of age.

The problems that early-term long-segment spinal fusion surgery might cause such as short stature, decrease in lung capacity, etc., are among the many topics discussed in the literature [3, 4]. Accordingly, some studies suggested the application of a short-term fusion after kyphectomy on the kyphosis site rather than the application of a long-segment fusion. However, this method turned out to be a more critical issue when compared to long spinal segment series because of its long immobilization period and recurrence of deformity even in the early term along with the failure of the correction [9, 10]. In the literature, Lindseth and Stelzer reported a loss of correction between 58 and 100% in patients to whom they applied kyphectomy and short-segment fixation with K-wire [11]. However, the literature indicates a low correction loss and a perfect correction rate in patients who underwent long-segment stabilization [12, 13]. In our study, eight of 24 patients got their implants removed, and three had correction loss. Implants of these three patients were removed due to infection, and the infection could be gotten under control after the removal. The correction loss rate was under 50% in these patients, and a revision surgery was not necessary for this rate. To sum up, implant failure is a critical complication in this patient group who underwent kyphectomy and short-segment fusion surgery. Long-segment instrumentation provides a stronger stabilization that prevents pseudoarthrosis and correction loss.

Wound site problems are observed in 83% of patients after short-segment stabilization [14]. The proximal parts of the spine are tend to form a flexion posture after short-term stabilization. As a result of the flexion posture, excessive tension of the implants causes irritation on the skin which leads to pressure sores. Some authors suggested the use of instrumentation which provided fixation from anterior in order to prevent skin problems [15]. However, this is a rather invasive procedure, and it does not allow pelvic fixation in rigid kyphosis surgeries which makes it biomechanically less reliable [16].

When we operate patients at young age, we need strong stabilization which does not prevent growth, limit lung capacity, and, at the same time, lead to an implant failure. Sliding growing rod technique was first applied to patients with early-onset scoliosis (EOS); however, we started to use this technique combined with kyphectomy in patients with MMC [4].

The polyaxial screws that we use in sliding growing rod technique have a sliding feature which gives us the opportunity of a strong fixation. The design of the system allows vertebral growth outside of the apex of the cephalic and caudal regions. In a study by Ouyand et al., the stability of the sliding growing rod technique was evaluated on sheep spine, the technique protected the stability as much as a totally fixated system also in movements such as flexion, extension, and lateral bending along with posterior instrumentation and fusion performance. In the literature, the outcomes of sliding growing double-rod technique are more successful with less complication rates when compared to the single growing rod system in patients with early-onset scoliosis and congenital scoliosis [17].

Pelvic fixation should also be applied to patients with thoracolumbar and lumbar MMC due to the nature of the deformity. In these patients, as kyphosis is in lumbar region, the vertebrae count is not enough to be able to support the stabilization. The system can fail due to the stabilization staying at the sacrum level and not being able to go down to pelvis. Iliac bone is mostly preferred for distal screw fixation due to soft structure of the sacrum [18]. Pelvic fixation plays a vital role in the treatment of the kyphosis in MMC which requires a mechanically better and more lever arm strength of the bone’s structural geometry and quality [5, 19]. To increase the kyphosis correction rate, pelvic fixation and its support on the sagittal balance are of vital importance. Niall et al. applied long-segment fixation to 24 patients by using different techniques and included pelvic stabilization in 10 of them. They concluded that patients applied pelvic stabilization had a better correction rate (52% against 64%) [20].

In our technique, we used iliac screws to strengthen the stabilization. We expected more stability and less morbidity with strong iliac screws that we used compared to the sacrum fixation.

In 2005, Akbarnia et al. reported an annual mean growth of 1.21 cm between T1 and S1 in patients that they operated by using growing rod technique due to early-onset scoliosis, and they indicated a similar rate to that of normal growth rate. However, they also claimed that those patients should be reoperated in every 6 months [21]. In 2015, Can et al. reported a growth of 1.05 cm between T1 and T12 in patients who underwent growing rod surgery in their study, and they reported a growth of 0.84 cm in patients who were operated with Luqué technique [22]. In another study, patients were operated with vertical expandable prosthetic titanium rib (VEPTR), and a growth rate of 1.81 cm between T1 and S1 was observed [23]. In our study, we found a growth rate of 0.74 cm between T1 and T12 and 0.77 cm between T1 and S1. Again, in another study conducted in 2020, we see that the amount of height growth was similar to that of our patients [22]. By this way, we believe that a second surgery will be needed at a way later stage if the rod that is left for growth is adjusted correctly, and we will be able to allow our patients to become adults without any deformity in their thoracal biology. Not to use binders is also of vital importance for our patients in the postoperative period for the patient comfort and prevention of pressure ulcers. In the literature, many authors needed to use binders for their patients to whom they applied classical growing rod technique or short-segment fusion [23].

Studies show that kyphosis has a corrective rate between 39 and 96%. The latter was mentioned in a study by Warner et al. in 1993 [25]. In another study conducted in 2016, VEPTR technique was first used in MMC patients, and 62% corrective rate was reported for kyphosis [24]. Many studies reported a kyphosis corrective rate of 41% in patients who underwent short-segment fusion [26, 27]. However, we observed a kyphosis corrective rate of 99.1% in our patients. This rate highlights the superiority of our technique (sliding growing rod) compared to short-segment fusion surgery [16, 23, 28].

Despite the many defined techniques, complication rates are often high [5, 29–31]. Average complication rates of the surgeries applied to patients with kyphosis associated with MMC are 50% regardless of the technique being either long-segment fusion or growing rod technique [16, 23, 28]. The most common complications were wound site problems and implant failures [30, 32]. In our study, we reported wound site problems and surgical site infections at a rate of 58.3%. Also, one or more debridement surgery was also reported at a rate of 50% (12) in our study. Kyphosis in lumbar area causes a thin skin and very weak subcutaneous tissues. During surgery, lordosis is formed in patients’ lumbar area out of kyphosis, so a critical emptiness is created in this area in which hematoma accumulates during the postoperative period. This is one of the many factors that increase the risks for site infection and skin necrosis. VAC systems are helpful in such conditions. We also applied VAC treatment to 9 (37.5%) patients in our study. Ozcan et al. [22], which include the average 27-month results of our patients, see that no implant removal surgery was performed in any patient. While all patients in that article were treated with VAC treatment and dressing in the early period, when the follow-up period of the patients increased to 53 months, it was observed that wound problems continued and implant removal surgery was needed in some patients. Even if we solve the wound closure problem in the early period, we should keep in mind that osteomyelitis may develop in the long term. Considering that the patients who underwent implant removal were treated with VAC due to discharge in the early period, we think that medical treatment can be given more carefully to prevent the development of osteomyelitis in these patient groups [22].

We used inverted “Y” incision in three patients due to their thin skin within the incision region and preoperative pressure wounds in order not to face wound site problems and to decrease the risk of dura damage during surgery. Thanks to this incision method, screw heads were mostly placed in the lumbar area with high muscle and fat tissue density. In these patients, we aimed to prevent pressure ulcers due to skin irritation caused by screw heads in the postoperative period [33].

Although our number of patients was inadequate for a statistically significant outcome, we believe that factors such as provided sagittal balance and high kyphosis corrective rate will decrease the possibility of postoperative complications. There are similar hypotheses in the literature [30, 31].

## Limitations

The limitations of our study were its retrospective nature and our relatively small number of patients. We observed that the overall number of patients who were applied kyphectomy surgery was low. In this regard, we believe that our patient number is similar to those in the literature, and our study also seems to be one of the most comprehensive works ever written. Many different surgery techniques were included in the literature; however, we observed that sliding growing rod technique provided an adequate stabilization for fusion after kyphectomy, and it allowed patients to grow. So, we believe that our technique is more beneficial for this patient group. We also did not observe preoperative malnutrition values and parameters together with positive urine culture which were all supposed to be evaluated; however, there are studies in the literature indicating a decrease in infection risk when two of these factors are considered and corrected in patient groups with high risk of developing an infection [34, 35]. Although, all of our patients had urological complications, we did not carry out any preoperative or postoperative urine culture in these patients which is also a limitation to our study. Another limitation of our study is the lack of having a proven method of measurement to objectively record and compare patient outcomes after spinal deformity surgery in patients with MMC. Hence, we are unable to objectively discuss the clinical effects of our outcomes on our patients.

## Conclusion

The treatment of kyphosis includes critical risks for MMC patients. We believe that the efforts for minimizing the risks in this patient group should be encouraged. We observed that kyphectomy and sliding growing rod technique was effective when applied together to the patient group with severe lumbar kyphosis associated with MMC in the correction of the deformity, adaptation to daily life, and improvement of sitting balance. This success is also sadly accompanied by growing number of complication rates. Patients and families were preoperatively informed in detail about high complication rates and repetitive multiple surgical procedures. Thankfully, we still accomplish preoperative targets even in patients who underwent multiple operations. In conclusion, we recommend the application of kyphectomy accompanied by sliding growing rod technique in this patient group, and yet we also believe more detailed series needed to decrease complication rates and to be able to evaluate long-term outcomes.
